# Mechanical and optimization studies of polypropylene hybrid biocomposites

**DOI:** 10.1038/s41598-022-06304-6

**Published:** 2022-02-15

**Authors:** Adeolu A. Adediran, Abayomi A. Akinwande, Oluwatosin A. Balogun, Oladele S. Bello, Miracle K. Akinbowale, Olanrewaju S. Adesina, Ayotunde A. Ojo

**Affiliations:** 1 Department of Mechanical Engineering, Landmark University, Omu-Aran, Kwara State Nigeria; 2 Department of Metallurgical and Materials Engineering, Federal University of Technology, Akure, Ondo State Nigeria; 3 Department of Mechanical Engineering, Ekiti State University, Ado Ekiti, Ekiti State Nigeria

**Keywords:** Materials science, Engineering, Mechanical engineering

## Abstract

Towards developing a polymeric matrix characterized by high strength to cost ratio, polypropylene (PP) was hybridized with low-cost particulate snail shell (PSS) and kenaf fiber (KF) via compression moulding at 180 °C and 0.2 MPa. The developed composites were grouped into three and labeled as mix 2, 4, and 10. Each group entailed the blend of 5, 10, 20, and 30 wt% KF with 2, 4, 10 wt% PSS respectively. From the results, it is observed that the hardness value was enhanced by the blend of 5 to 30 wt% KF and 2, 4, and 10 wt% PSS. However, 2 wt% PSS mix with 5 to 30 wt% KF resulted in progressive improvement in impact, compressive, flexural, and tensile strengths values. The 4 wt% PSS yielded consecutive increase in impact, compressive and flexural strength when combined with 5 and 10 wt% KF. However, it was observed that subsequent addition of 20 and 30 wt% KF led to a marginal reduction in the strength values. The tensile strength attained optimum value when 4 wt% PSS was commixed with 30 wt% KF. Conversely, the combinations of 10 wt% PSS with 5, 10, 20, and 30 wt% KF had no significant improvement to the mechanical properties of PSS/KF-bio-PP composite (except for hardness) siring strength decrease. Taguchi optimization revealed that the collage of 4 wt% PSS and 10 wt% KF presented optimum mix for hybrid bio-PP composite.

## Introduction

Fundamental engineering materials are in most cases classified as metals, ceramics, and polymers. These classes of material find application in various aspect of engineering and the choice are based on their properties. The selection of one class of material over another is on account of comparative advantage in terms of property and cost. Metals are characterized by high strength, ductility, and closed packed structures^1^ while ceramics are hard, have good heat and corrosion resistance, dense and brittle^2^. Polymer, on the other hand, is light in terms of weight, possesses a high strength-to-weight ratio, low cost, and has resistance to corrosion^3^. Nowadays, polymer has found successful engagement in most engineering applications as substitutes to metals because of cost implication and corrosion resistance^4^. Engineering polymers are employed in biomedical and biomimetics, aerospace, automobile, electricity and electronics, structures, clothing, and material development^5,6^. Polymeric materials are further classified into thermoplastics, thermosets, and elastomers. Thermoplastics like polypropylene, polyethylene, and polyvinyl chloride have high demand based on their everyday use. Polypropylene (PP) for instance, is applied in packaging, automobile, textile, medical and other forms of application^7, 8^. Being an engineering material, it has good chemical and corrosion resistance, it is tough, and possesses good fatigue and heat resistance^9^. According to Maddah^10^, energy-saving policy in automobile manufacturing necessitates the use of lightweight engineering materials for fuel optimization. Based on this and owing to the lighter weight of PP when compared with most polymeric materials, PP is being used for different parts of the vehicle. Some of the areas of use in cars are dashboards, batteries, AC ducts, doors, and quarter panels^11–13^. Also, thermoplastic olefin produced from PP is utilized in car bumpers, air drums, and rocket panels^14,15^. In aerospace, PP is employed for sandwich composite panels while in structures, it has application as fibers for reinforcing concrete^16,17^. For better performance in service, base polymeric matrix undergoes structural alteration and modification via different processes one of which is reinforcement with different materials; infusing synthetic and agro by-products in the matrix. Agro-by-product reinforcement of polymer matrix in form of bio fillers and bio fibers are encouraged based on their low cost and reuse. The agro-based products cause some modification at the microstructural level bringing about enhanced property of base material. Authors Onuegbu and Igwe^18^ investigated the effect of snail shell powder on the properties of PP. The snail shell powder was sieved to 15 µm, 30 µm, and 42 µm after which they were added at varying proportions of 10, 20, 30, and 40% respectively. The mechanical properties were assessed and the result revealed progressive appreciation in tensile, impact, and flexural strengths. Analysis of the results showed a higher improvement of properties with reduced particle sizes. Optimum mechanical properties were attained at 40% of the powder for all particle sizes considered with 15 µm having the highest performance. Studies from Onuegbu and Nwanonenyi^19^ examined the mechanical properties of PP by adding pulverized groundnut husk at 2, 4, and 6%. Particle sizes were varied at 0.2, 0.4, 0.6, 0.8, and 1 mm. The result showed enhancement in tensile, flexural, and impact strengths on account of adding up to 6 wt% of the powder. From the outcome, the inclusion of filler contributed to the property improvement of the PP matrix. On the other hand, coconut shell particles have been reported as a strengthener in the production of polymer matrix composites^20^. Varying weight percent of coconut shell particles at 10, 20, 30, and 40 wt% were utilized in the production of polymer-based composites. The results revealed that irrespective of size, an increasing proportion of the particle improved the yield, tensile and flexural strengths even as hardness was enhanced^20^. In recent times, cardanol was used with untreated and NaOH treated coir particles at different sizes of 25, 50, and 75 µm ^21^. Their studies revealed an enhancement in tensile, flexural, and impact strengths respectively. Further observation revealed that 25 µm treated coir particles performed optimally. Equally, 3, 6, 9, 12, and 15 wt% snail shell powder were observed to improve tensile strength in recycled waste plastic developed for automobile application^12^. Similarly, compressive, impact, and flexural strengths, and hardness were enhanced by the blend of 10, 20, 30, 40, and 50 wt% of carbonized eggshell filler^22^. Researchers also investigated the influence of agro-fiber as reinforcement in polymers reporting various outcomes. Natural fibers like sisal, banana, and coir were employed in reinforcing epoxy resin in the literature^23^. Tensile strengths, flexural strengths, and hardness of the epoxy composites were enhanced by the fibers. Another common fiber is bagasse^24^. Authors Akinwande et al.^25^ examined the effect of proportion and length of coir fiber in PP towards property enhancement for automobile application. Experimental results depicted that the inclusion of fiber at 5 to 20% played a major role in improving tensile and flexural properties. Impact resistance was enhanced up to 25 wt% of fiber while hardness showed progressive improvement with coir fiber fraction of 5 to 30 wt%. Optimization engaged in via response surface methodology denoted fiber length 34.85 mm at 25.76% proportion is most desirable. Kenaf/polypropylene composite was developed by authors Lee et al.^26^. As observed, 10, 15, 20, and 25 wt% kenaf fiber-enhanced tensile and flexural properties over neat PP depicting the fact that kenaf fibers in PP ensued positive modification of mechanical properties for engineering application. From the findings of Ramli et al.^27^, PP was reinforced with oil palm fiber matrix. From the outcome, 40 wt% fiber reinforced PP showed better properties than control PP matrix with regards to flexural strength, tensile and flexural moduli. Jute fiber employed by Kabir et al.^28^, led to the enhancement of tensile and flexural properties. From these literatures, it’s clear that agro-by products in terms of fiber, play major role in the improvement of mechanical properties of developed polymeric composites. Additionally, hybrid polymer composites of fiber and particulate fillers blend had been a subject of investigation for years; as investigated by Balaji et al.^29^ which entailed a study on bagasse fiber/coconut shell hybrid biocomposites. On the other hand, hybridized polypropylene with coir fiber and yam peel particulate as reported by Adediran et al.^30^, showed an improvement in impact strength as well as tensile and flexural properties. In Adeosun et al.’s work^31^, hybrid polyester composite with the blend of coconut and snail shell particulate in unsaturated polyester was reported. By contrast, coconut shell particulate outperformed snail shell particulate especially tensile strength and Brinell hardness, while snail shell outperformed coconut shell with regards to bending and impact strengths. Generally, optimum results were obtained with portions between 10 and 20 wt% for the two particulates proving that dual particulate contributes to property enhancement of polymers. It is well known that, agro-fibers and particulate fillers have a promising future as reinforcement in polymers. This study, therefore, involved property evaluation of polypropylene matrix reinforced with kenaf fiber and particulate snail shell. The choice of polypropylene is on the dint of low cost and its availability in the Nigerian market. Improving low-cost PP matrix with agro-byproduct can lead to the development of useful engineering products which are affordable. In addition to the property evaluation, statistical analysis was carried out on obtained results to derive an optimum combination of kenaf fiber and particulate snail shell.

## Materials and methods

### Materials

**Figure 1:**
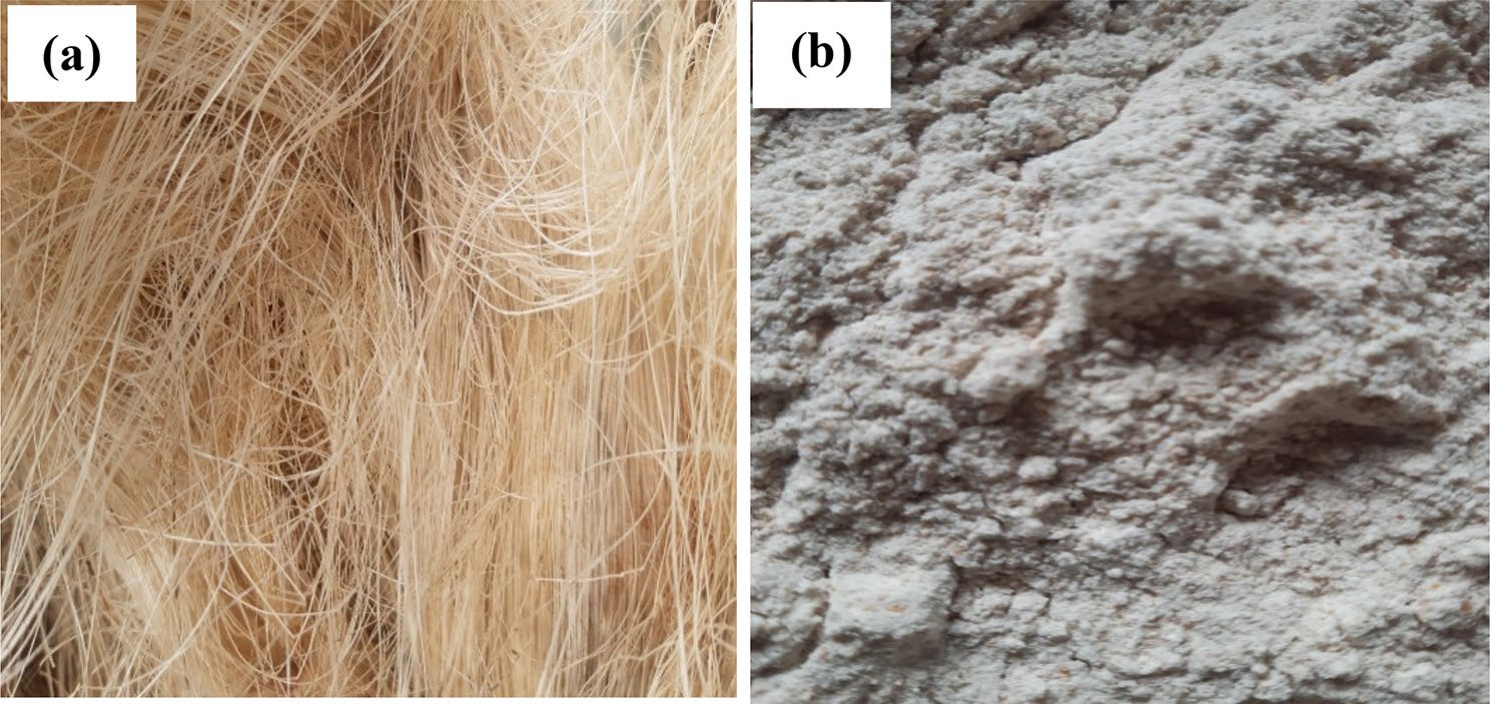
Image of input materials (**a**) kenaf fiber (**b**) Particulate snail shell.

**Figure 2:**
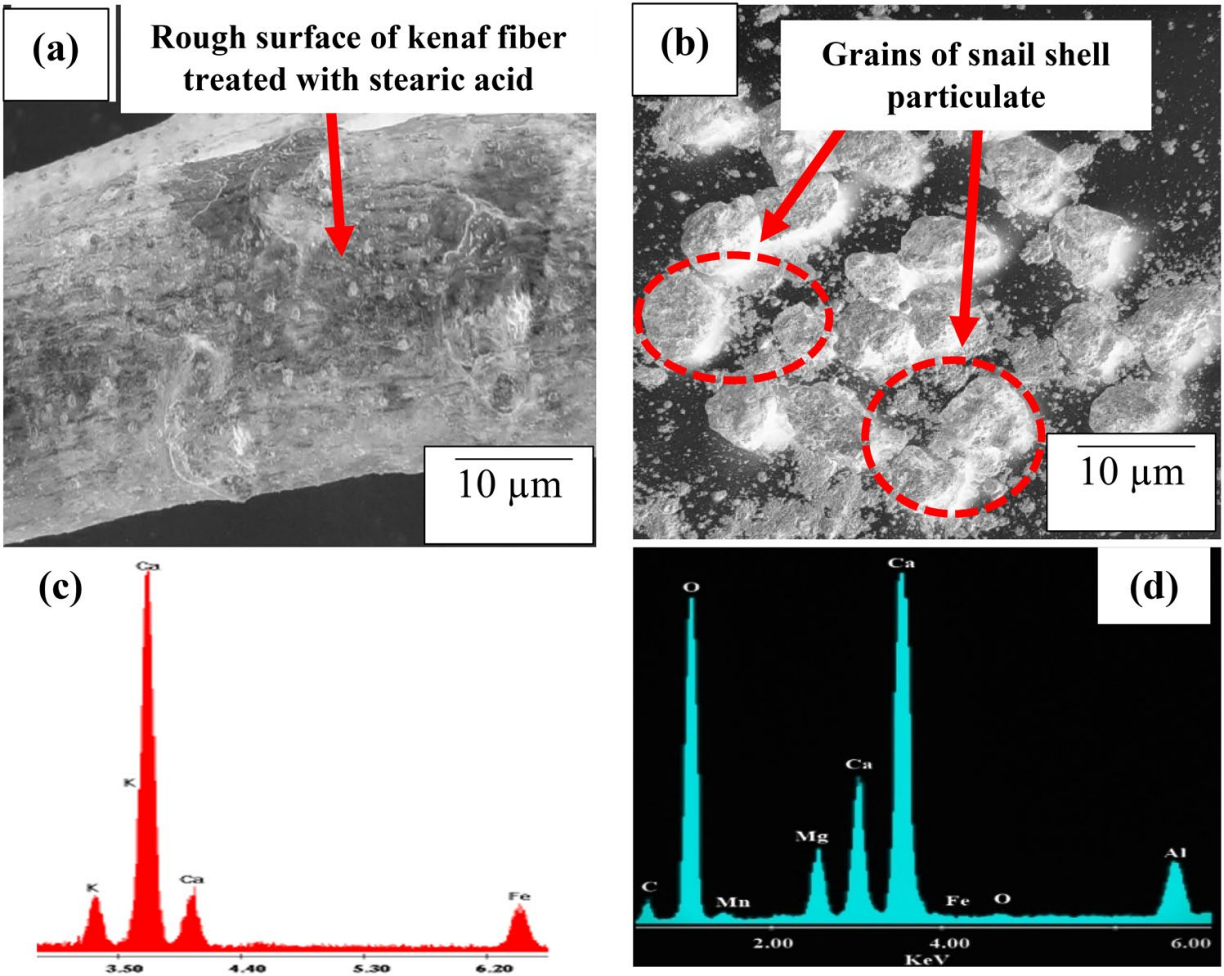
Morphological images of input materials (**a**) kenaf fiber (**b**) Particulate snail shell and EDX spectra for (**c**) kenaf fiber (**d**) Particulate snail shell.

Input materials engaged in this research are stearic acid, kenaf fiber, snail (African giant snail) shell, and virgin polypropylene (PP) pellet. Kenaf fiber and snail shell were procured from a nearby farm in Omu-Aran, Kwara State, Nigeria while stearic acid and virgin PP pellet was procured from a merchant in Omu Aran, Kwara State. Procured kenaf fiber was washed in warm water to removed attached impurities and sundried for 2 days. Afterwards, the fiber was cut to 25 cm length and treated with 0.8% stearic acid according to procedures followed by Salman et al.^32^ where surface treatment of kenaf fiber with 0.8% achieved optimum performance. Treated fiber (Fig. 1a), was washed with distilled water and oven-dried at 60 °C for 8 h. The snail shells were cleansed in warm water to remove impurities present and sun dried for 3 days after which the dried shells were crushed and pulverized followed by sieving using a laboratory electric sieve shaker. Snail shell particulate sieved to − 25 µm (Fig. 1b), were collected, oven-dried at 50 °C for 2 h. 25 µm size was adopted on account of observations noted in Udhayasankar et al.^21^. Figure 2a–d show the morphological images and energy dispersive spectra of the input materials. The chemical constituents of treated kenaf fiber used in the study is as presented in Table 1 while Table 2 shows the chemical composition of the particulate snail shell.

**Table 1:** Chemical constituents of treated kenaf fiber used in the study.

Chemical constituents	Cellulose	Hemicellulose	Lignin	Moisture	Ash	Others
Amount	52.3	22.4	7.9	6.4	3.4	7.8

**Table 2:** Chemical composition of particulate snail shell employed in the present investigation.

Compound	Amount
CaO	59.8
SiO_2_	0.89
Al_2_O_3_	0.16
Fe_2_O_3_	0.78
MnO	0.06
MgO	0.96
Na_2_O	1.05
Others	4.81
Loss on ignition	31.33

Surface treatment of kenaf fiber revealed a rough surface of the fiber occasioned on account of the removal of wax and impurities. The process also led to a reduction in lignin and hemicellulose content (Table 1), thereby decreasing the hydrophilic tendency of the fiber. Figure 2c highlights the EDX spectrum of KF from which it is noted that calcium (Ca) showed a high peak, suggesting the presence of calcium-based compound which contributes to the strength enhancement of PP matrix. Figure 2b reveals the microstructural features of PSS. The shape of the particles is mostly spherical of which the shapes promoted infilling of pores in matrix reducing inter-particle distance, effect of which reflected in property enhancement. From Fig. 2d, peak showing calcium (Ca) and oxygen (O) are observed to be higher than other elements. The resultant effect of the Ca and O presence shows the presence of CaO as confirmed in Table 2, contributing to the strength and hardness of composites when PSS was imbued in the PP matrix.

### Mix proportion

The development process involved blending of particulate snail shell (PSS) and kenaf fiber (KF) in polypropylene (PP) matrix at varying proportions using waring laboratory blender (8011EG). The control mix was produced as pure polypropylene (PP) with no KF and PSS admix. Three group mixes of samples were considered and are labeled as mix 2 composites for the first group, mix 4 composites for the second group and mix 10 composites for the third group. Group labeled mix 2 composites entailed the blend of 0, 5, 10, 20, and 30% KF by weight of PP with 2 wt. PSS. Similarly, groups labeled mix 4 composites consist of the blend of 4 wt% PSS and 0, 5, 10, 20, and 30% KF and mix 10 composites contained the blend of the same proportion of KF and 10% constant PSS weight fraction. 2, 4, and 10 wt% PSS were considered based on conclusion made by Chris-Okafor et al.^33^ in which mechanical property optimization of low-density polyethylene matrix was realized by the incorporation of 2 to 10 wt% snail shell powder.

### Production process

Compression moulding machine (NG-BU-P11) operated at a temperature of 180 °C and pressure of 0.2 kPa for 10 min on each sample was employed. 180 °C was adopted because temperature trials above 180 °C produced burnt samples and below produced less compacted samples. Dump bell shape mould 150 mm length and 3 mm thickness was used in the preparation of samples for the tensile test. Samples for flexural and impact were prepared using a mould of dimension 150 × 50 × 3 (mm^3^) and 63.5 × 12.7 × 2.5 (mm^3^) according to Adediran et al.^34^. Samples for compressive strength were produced in moulds 40 mm diameter and 80 mm length. Wax was robbed in the mould cavity while the top and base of the mould were covered with Teflon sheets before compression. Samples were left to cure for 24 h sequel to careful removal from moulds and cleaning.

### Property characterization

#### Izod impact strength

Izod impact strength was appraised by subjecting notched samples (V-shaped) to impact employing Hounsfield balance impact tester (3915) in line with ASTM D 256-10^35^. The pendulum of the tester was set at 165° to fracture samples and impact energy to fracture was measured. An average of three samples was recorded for each mix proportion.

#### Tensile and flexural strength

A universal testing machine (Instron 3369 series) was used in the test in line with ASTM D 3039 M-17^36^ applying a load of 100 N at a crosshead speed of 5 mm/min. Flexural samples were subjected to a three-point bending load of 1 kN. A crosshead speed of 10 mm/min was adapted and a span length of 100 mm was maintained for all samples. The test was done in accordance with the ASTM D 790–17^37^ procedure. An average of three samples were noted for each mix proportion.

#### Hardness

The hardness of the developed composite was probed as par ASTM D 2240-15e1^38^ employing shore Durometer apparatus. The samples were indented five times on the surface and the average value was obtained.

#### Compressive strength

Quasi-static compression was examined on cylindrical samples produced as prescribed by ASTM D 695-15^39^. Samples were subjected to compressive load at a strain rate of 0.1 s^−1^ and piston speed of 0.25 m/s. An average of three samples were recorded for each mix proportion.

#### Microstructural analysis

Microstructural analysis was carried out with the use of High magnification fluorescence microscope (E-807Y) operated at 5.0 kV and magnification of × 5000.

## Results and discussion

### Microstructural and property evaluation

**Figure 3:**
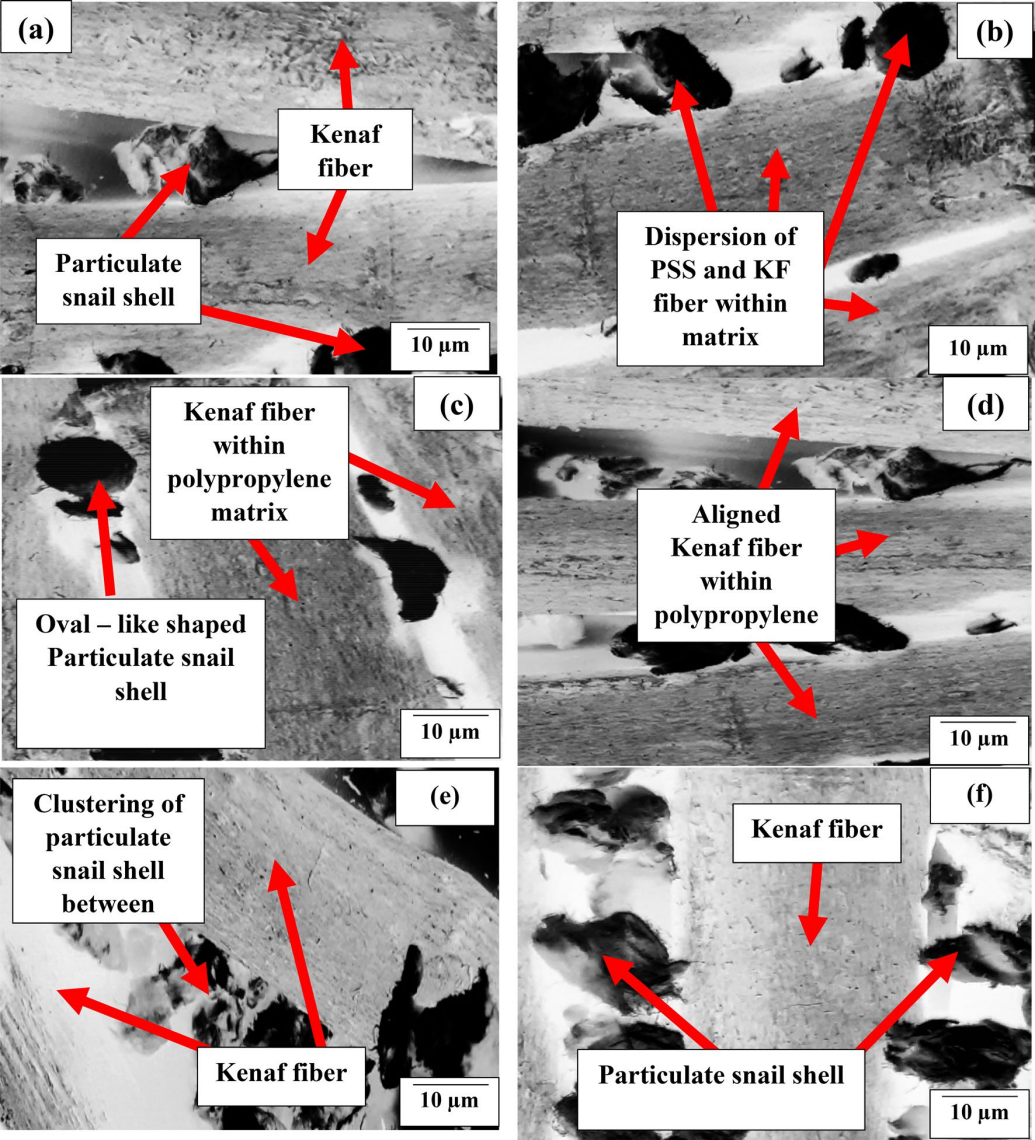

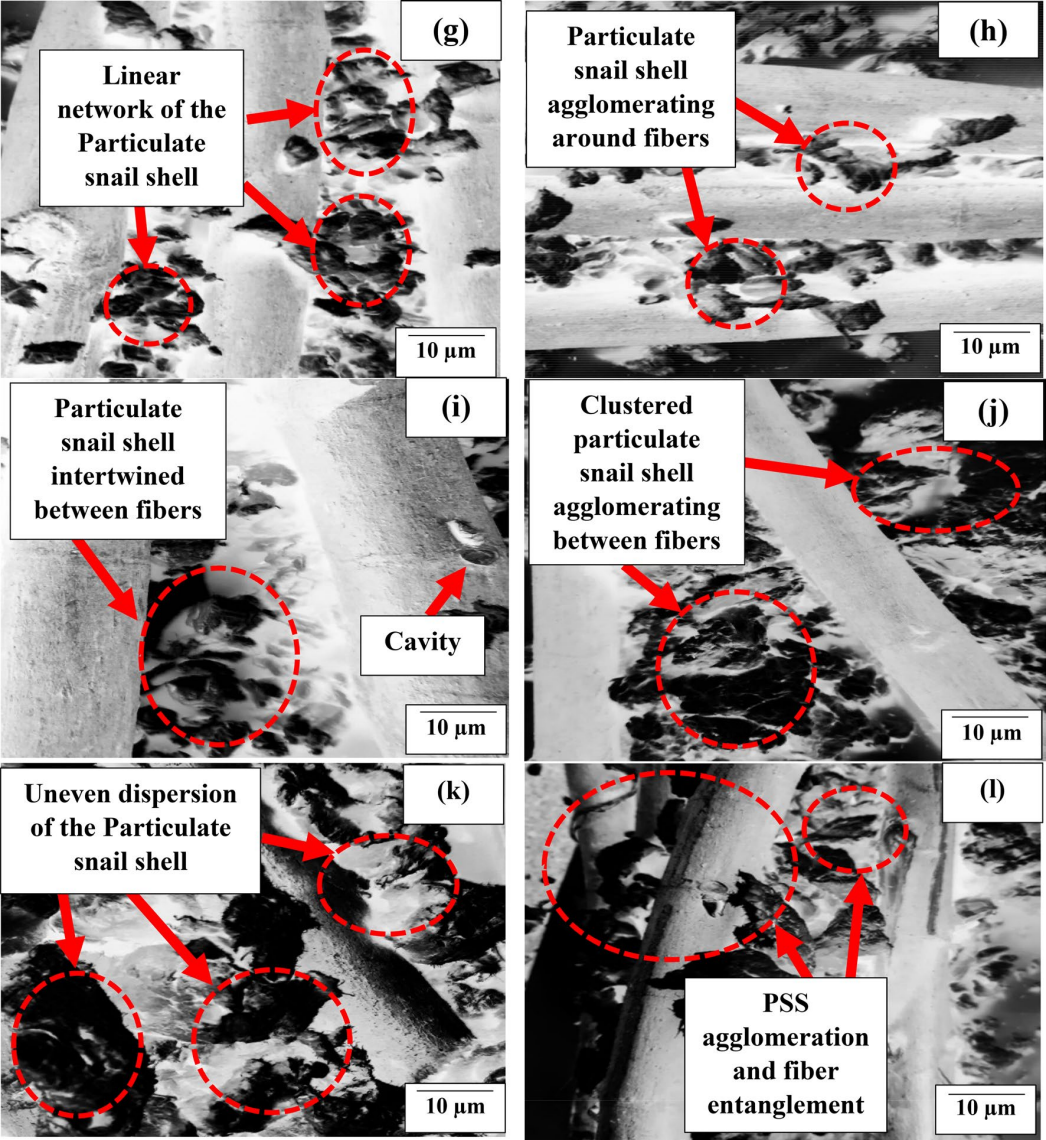
Microstructural image of polypropylene-PSS/KF composites developed, (**a**–**d**) reflects the images of mix 2 composites; (**e**–**h**) show the mix 5 composites containing 5 wt% of the particulate; (**i**–**l**) present morphological representation of the mix 10 composite prepared with 10 wt% PSS.

Figure 3 presents the microstructural images of the fibers in the composites developed.

Figure 3a–d reflects the images of mix 2 composites, it was observed that there is even dispersion of PSS around the fiber which led to even stress transfer between PSS and KF even under deformation. This observation is typified in the investigation carried out by Balaji et al.^29^ and Anuar et al.^46^. In their study, the inclusion of kenaf fiber at 5 to 20 wt% in maleic anhydride grafted polypropylene led to enhancement of strength of composites. Additionally, kenaf fiber of 10, 20, 30, and 40 wt% was observed to boast composites strength in Salman et al.^32^, though contrary observation was reported in Kim and Cho^47^ where 10, 20, and 30 wt% kenaf fiber manifested a progressive decrease in strength. Figure 3e–h depict the mix 5 composites containing 5 wt% of the particulate. Figure 3e portrays agglomeration of PSS between kenaf fiber serving as point of stress concentration. Figure 3f reflects the particulate dispersion and interaction with kenaf fiber within matrix enhancing bond strength. This is held responsible for improved performance in the strength properties. Figure 3g presents particulate agglomeration around fiber serving as points of agglomeration, affecting the strength performance negatively. Figure 3h displays how the particulate are displayed along the fibers, depicting reasons for improved strength. Meanwhile, Fig. 3i–l present morphological representation of the mix 10 composite prepared with 10 wt% PSS. The major feature observed is the agglomeration of particles appears alongside the fibers. This is responsible for low performance of the mix 10 samples.

#### Hardness response of polypropylene-PSS/KF composites

**Figure 4:**
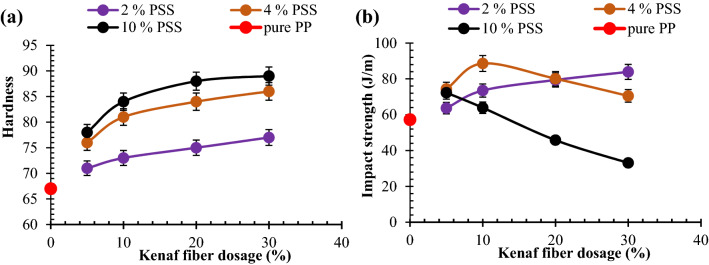
Graphical plot for the interaction between admix of kenaf fiber (KF)/particulate snail shell (PSS) at varying proportion and (**a**) hardness (**b**) impact strength.

As observed in Fig. 4a, hardness was enhanced by the introduction of KF in the PP matrix. Mix 2 composites group entailed blend of 2 wt% PSS and 5, 10, 20, and 30 wt% KF. It was noted that increasing KF loading amounted to the enhancement of hardness. This trend is based on the interlink between fiber and matrix enhanced by alkaline treatment of the fiber which promoted interfacial adhesion between the fiber and polypropylene matrix. A similar finding from the investigation of Balaji et al.^29^ portrayed an uptrend in hardness with increasing banana fiber loading of 5, 10, 15, and 20 wt% in epoxy matrix; corroborating the outcome of this study.

From the result obtained, the interaction between the KF and PSS led to the enhancement of hardness. It was reported that Shore D hardness showed an enhancement as fiber content increased from 0 to 20 wt%^40,41^. Similarly, Durowaye et al.^42^ corroborated the findings of this study as date palm fiber loading showed improvement of hardness in polyester composite developed in their investigation. Findings from Swain and Biswas^43^ and Chelliah^44^ linked the improvement in hardness to the cross-link of fiber with the polymer chains resulting in a complex network.

Present study revealed infusion of 5, 10, 20, and 30 wt% KF in the PP matrix depicted 6.0, 9.0, 11.9, and 14.9% uptrend in the value with respect to the pure PP (control sample). This delineates the fact that inclusion of kenaf fiber with PSS in polypropylene matrix ensues improvement in hardness. In the case of mix 4 composites (4 wt% PSS) which involved commix of 4 wt% PSS at the constant amount and varying KF dosage of 5, 10, 20, and 30 wt%, it is revealed that increasing KF portion resulted in the progressive rise in values. As highlighted in Fig. 4a, intermix of 4 wt% PSS further promoted hardness since values reported for mix 4 composites are higher than the values of mix 2 composites. This showed that the presence of PSS also enhanced hardness which is associated with the CaO presence of the particulate (Table 3 and Fig. 2d). Furthermore, hard snail shell particulate presence in PP matrix led to close packing thereby enhancing cohesion between particles consequence of which led to boosting the property. The Coalesce of 5, 10, 20, 30 wt% KF and 4 wt% PSS offered 9.0, 16.4, 17.9, and 17.9% boast to hardness; giving higher value than the mix 2 counterpart. The observation corroborates the study carried out by Nwanonenyi et al.^45^ which explicated enhancement of hardness of low-density polyethylene matrix by the reason of adding periwinkle shell particles in the dosage of 2 to 16 wt%.

**Table 3:** ANOVA for harness.

Source	DF	Adj SS	Adj MS	F-value	*P* value
A	1	168.69	168.686	12.88	0.005
B	1	391.11	391.109	29.86	0.000
A*A	1	26.17	26.172	2.00	0.188
B*B	1	63.90	63.903	4.88	0.052
A*B	1	306.22	306.219	23.38	0.001
Error	10	130.98	13.098		
Total	15	2178.93			

Figure 4a also show values for mix 10 composites (10 wt% PSS) which contained the blend of 10 wt% particulate snail shell at a fixed amount and 5, 10, 20, 30 wt% KF; the result depicted an uptrend in hardness with fiber loading. In comparison with values of mix 4 composites, mix 10 composites exhibited higher hardness values at each proportion of kenaf fiber mix. Commix of 10 wt% PSS and 5, 10, 20, 30 wt% KF amounted to 13.4, 23.9, 26.9, and 32.8% respectively over pure PP.

### Impact resistance response of polypropylene-PSS/KF composites

Figure 4b highlights the relationship between impact strength and KF/PSS dosage. Kenaf fiber dosage of 5, 10, 20, and 30 wt% in the presence of 2 wt% PSS demonstrated uptrend in impact strength; yielding 11.1, 28.3, 38.6, and 46.4% upgrade respectively relative to the value of pure PP. The result can be traced to enhanced interfacial adhesion between fiber and matrix promoting even stress distribution. Furthermore, as observed in Fig. 4b, admixing of 4 wt% particulate snail shell and kenaf fiber at 5 and 10 wt%, as depicted in mix 4 composites, revealed further enhancement of strength by 29.8 and 52.6% respectively relative to pure PP. The attainment is based on two factors, one of which is the interlocking of the fiber with matrix chain promoting resistance to high strain rate coupled with even distribution of the fibers. Second is the even dispersion of the PSS particulate within the matrix enhancing reinforcement/matrix interaction. Figure 3e,f identified the image of samples doped with 4 wt% PSS and 5 and 10% KF respectively. There is particulate  dispersion within the matrix which contributed to interaction between the reinforcement and matrix. This is further boosted by the arrest of cracks by the fibers. Further intermix of 4 wt% PSS with 20 and 30 wt% KF resulted in a 7.0 and 17.5% reduction in strength relative to the value obtained when 4 wt% PSS and 10 wt% KF was infused; though the value was still an enhancement of 43.8 and 27.6% over pure PP. Figure 3g,h depicts points of fiber entanglement and PSS clustering/agglomeration owing to larger volume fraction occupied by the two inputs which may cause segregation and agglomeration. These features serve as points of stress concentration eventually reducing the strength of the materials.

For mix 10 composites which were doped with 10 wt% fixed proportion of PSS, it is evident from Fig. 4b that 5, 10, 15, and 20 wt% KF had progressive reduction in strength. The reason for this is stress concentration as a result of fiber entanglement and particulate agglomeration between fibers as revealed in Fig. 3i–l. Therefore, collage of 10% PSS and 5, 10, 20, and 30 wt% KF demonstrated a negative influence on impact strength. Meanwhile, peak impact strength was achieved at intermix of 4 wt% KF and 10 wt% PSS.

With respect to the findings from Daramola et al.^48^, 44.4% optimum enhancement of impact strength was observed with the use of chitosan microparticles (3.5 µm average diameter) whereas in present study, optimum enhancement of 54.4% enhancement was realized for the same strength. Likewise, optimum impact strength enhancement attained in present study is superior to 16.7% improvement realized in Husseinsyah et al.^49^ when 10 wt% chitosan filler was added to polypropylene matrix. Comparative result of Amri et al.^50^ reflected 20% optimum enhancement in impact strength of polypropylene matrix when 10 wt% chitosan was added, a value lower than optimum improvement realized in this study.

#### Compressive strength of polypropylene-PSS/KF composites

Few studies carried out on compressive strength of polymer composites has shown compelling outcomes. The report from Dash^51^, revealed that the mix of banana fiber in epoxy at 5 and 10% vol. fraction fortified the epoxy composites containing teak wood dust. It was noted that 5 and 10% banana fiber yielded enhancement of 7 and 14.9% in compressive strength at 10% teak wood dust. According to the study, 20% fixed teak wood dust combining with 5 and 10% banana fiber, kindled 5% reduction in and 13.1% increase in compressive strength respectively. Similarly, study of Adediran et al.^34^, noted that an increase in dosage of bamboo fiber resulted in an insignificant contribution to the compressive strength while particulate distribution in matrix unfurled significant effect on the strength.

**Figure 5:**
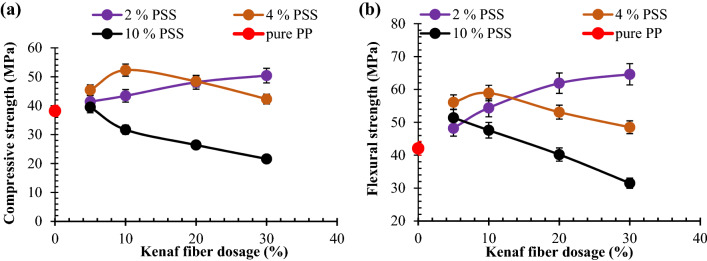
Graphical plot for the interaction between admix of kenaf fiber (KF)/particulate snail shell (PSS) at varying proportion and (**a**) compressive strength (**b**) flexural strength.

In this study, Fig. 5a revealed that 5 to 20 wt% KF loading in mix 2 composites had an uptrend in compressive strength, amounting to an increment of 8.3, 13.6, 23.1, and 27.5% over neat PP. The progression is ascribed to the enhanced stress distribution based on the fiber presence. The influence of kenaf fiber on compressive strength was further boasted on inoculation of 4 wt% PSS in the matrix (mix 4 composites) as revealed in Fig. 5a. The 5 and 10 wt% KF yielded 18.8 and 36.9% improvement over pure PP accordingly. The implication of this is that coalesce of 4 wt% PSS and 5; 10 wt.% KF enhanced compressive strength owing to CaCO_3_ presence in PSS which contributed to strength value. Also, the ensuing close packing of particles and fiber interlock with polymer chains resulted in cohesive interaction between particles and fiber, engendering even stress propagation as obtained in Fig. 3e,f respectively. It is noteworthy that coagulation of PSS and fiber entanglement in Fig. 3g,h is associated with depreciation in strength when 20 and 30 wt% KF is mixed with 4 wt% PSS in PP matrix. Thus, yielding 7.4 and 19.1 decrease in values obtained at 10 wt% KF/4 wt% PSS. The coexistence of 4 wt% PSS/10 wt% KF in PP matrix had an improvement of 15.2% over values on the infusion of 4 wt% PSS/5 wt% KF. As observed in Fig. 5a of this work, 4 wt% PSS/10 wt% fiber ensued strength increase of 36.9% over neat PP. Intermix of 5, 10, 20, and 30 wt% kenaf fiber and 10 wt% PSS led to a reduction in compressive strength (Fig. 5a) on account of coagulation of particles as revealed in Fig. 3i–l respectively. The infuse of 5, 10, 20, and 40 wt% KF in mix 10 composites led to 13.3, 21.7, 12.8, and 3.7% depreciation in strength. The maximum strength was attained at 10 wt% PSS/4 wt% KF in PP matrix as indicated in Fig. 5a.

#### Flexural response of polypropylene-PSS/KF composites

Natural fibers have proved to be good agents in improving the flexural properties of polymers^29,52–54^. Findings from Sushanta^55^ revealed progressive enhancement of flexural strength of PP matrix reinforced with banana/glass fibers corroborating the observations reported in the present study. From Mir et al.’s study, kenaf fiber inclusion (KF) resulted in strength enhancement for KF in the proportion of 5, 10, 20, and 30 wt%.

In the present investigation, the presence of KF in mix 2 composites reflected a consecutive rise in flexural strength with fiber loading which occurred on the dint of fiber-matrix chain network formed, coupled with enhanced adhesion between KF and PP matrix (Fig. 5b). These proportion yielded 14.4, 29.2, 47.0, and 53.4% improvement in flexural strength respectively, with respect to pure PP^41^. It is evident that the evaluation of mix 4 composites (4 wt% PSS) displayed enhancement in flexural strength on the infusion of 5 and 10 wt% KF. In contrast, subsequent addition of 20 and 30 wt% KF amounted to 7.8 and 16.2% decline relative to 10 wt% KF value. In comparison, mix 4 composite samples blended with 4 wt% (PSS) performed better than mix 2 counterparts (2 wt% PSS) when 5 and 10 wt% KF was infused. This is based on enhanced interparticle cohesion owing to closed packing afforded by particulate presence.

Particulate addition has been reported in some studies to enhance flexural strength as noted by Obasi^56^. It was reported that incorporation of 0, 5, 10, 15, 20, and 25 wt% peanut husk powder in low-density polyethylene resulted in the progressive rise in flexural strength with 25 wt% of the powder kindling up to 63.3% enhancement. Similarly, findings of Asuke et al.^57^ revealed the same trend when 0, 5, 10, 15, 20, and 25 wt% of carbonized and uncarbonized fish bone powder was infused into polypropylene matrix. At 25 wt%, enhancement of 53.8 and 35. 9% were realized for both carbonized and uncarbonized respectively. These reports are in tandem with the findings showcased in the present study.

As noted in Fig. 5b, for mix 4 (4 wt% PSS) mix, blend of 4 wt% PSS and 5, 10, 20 and 30 wt% KF showed progressive reduction in strength. Meanwhile with respect to flexural strength of neat PP, 5 and 10 wt% KF dosage instigated 22.1 and 13.1% increase in strength while additional dose of 20 and 30 wt% KF in the blend led to 5.4 and 25.2% decrease in strength value when compared with the value of neat PP. The aforementioned observations are hinged on agglomeration of the PSS particles and entanglement of fiber as obtained in Fig. 3i–l respectively. Maximum flexural strength is attained when 10 wt% KF is co-blended with 4 wt% PSS which is based on the interplay between kenaf fiber which serves as crack arrester and particulate snail shell which enhanced cohesion between particles and also played a major role in impeding dislocation movement. Shyang et al.^58^ studied the effect of natural hydroxyapatite on mechanical performance of poly (methyl methacrylate) biocomposites. Hydroxyapatite content from 5 to 20 wt% ensued progressive reduction in flexural strength and strain contrary to observation realized in this study.

#### Response of polypropylene-PSS/KF composites to tensional stress

**Figure 6:**
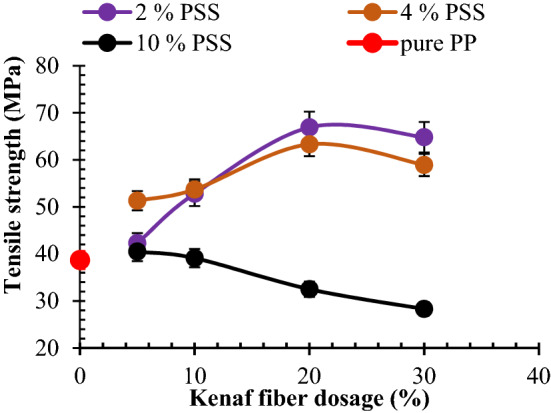
Graphical plot for the interaction between admix of kenaf fiber (KF)/particulate snail shell (PSS) at varying proportion for the Tensile strength.

The result of tensile strength against KF/PSS is as presented in Fig. 6. Evaluation of mix 2 composites (2 wt% PSS) exhibited a steady increase in strength with KF loading up to 20 wt%. This observation is engendered by interfacial adhesion between filler and matrix coupled with fiber dispersion in matrix enhancing stretching. Similarly, there is a strong interlink between fibers and the polymer chains. Findings from Haque et al.^59^, Haque et al.^60^, Haque and Islam^61^ revealed that fiber inclusion in polymer matrix up to 20 wt% ensued enhancement of tensile strength. Findings from Balaji et al.^29^ achieved optimum tensile strength at 15 wt% banana fiber an increase of 65 and 68.9% over neat epoxy, for 10 mm and 20 mm length of the fiber respectively.

Meanwhile, kenaf fiber employed in present study had an optimum tensile at 20 wt%, an enhancement of 72.9% over neat PP. It is noteworthy that 30 wt% KF had a slight reduction of 3.2% in strength compared with value attained at 20 wt% KF. The reduction is fraternized by possible reduced stretching of fibers owing to frictional interaction between the two input materials. 5, 10, 20, and 30 wt% KF resulted in 11.0, 36.4, 72.9, and 62.3%.

Evidently, in mix 4 composites (4 wt% PSS), it is observed that KF loading demonstrated an uptrend in tensile strength from 5 to 20 wt%. Further addition of 30 wt% KF manifested slight depreciation in strength. By comparison, the tensile strength of mix 4 composites reinforced with 5 and 10 wt% KF yielded 8.5 and 3.9% lesser than their counterpart in mix 2 depicting the fact that the coalesce of 2 wt% PSS and 5 and 10 wt% KF amounts to strong cohesion and even stress distribution within the PP matrix as observed in Fig. 3a,b. Mixing of 4 wt% PSS with 20 and 30 wt% KF evoked 4.2 and 13.0% decrease in tensile strength as compared with their counterpart in mix 2. As highlighted in Fig. 6 concerning mix 10 composites (10 wt% PSS), the mix of 5 to 20 wt% KF had a decrease in strength for 5 to 20 wt% based on a corollary of PSS coagulation and fiber entanglement as noted in Fig. 3i–l respectively. Evaluating performance of PSS on tensile strength; in general, PSS addition performed low on tensile strength in that its presence led to a decrease in strength (apart from when 4% PSS was co-blended with 5 and 10 wt% KF). The poor performance of PSS on tensile strength is reported by Okafor et al.^62^ in which 5, 10, 15, 20, and 25 wt% led to the progressive decrease in strength associated with possible stress concentration at particulate/matrix boundary. By contrast, the 10 wt% chitosan microparticle from Daramola et al.^48^ yielded ~ 30% improvement in tensile strength and compared with optimum enhancement of 72.9% in present study. The discrepancy may be on account of kenaf fiber distribution within matrix which aided stress distribution.

### Statistical analysis

**Figure 7:**
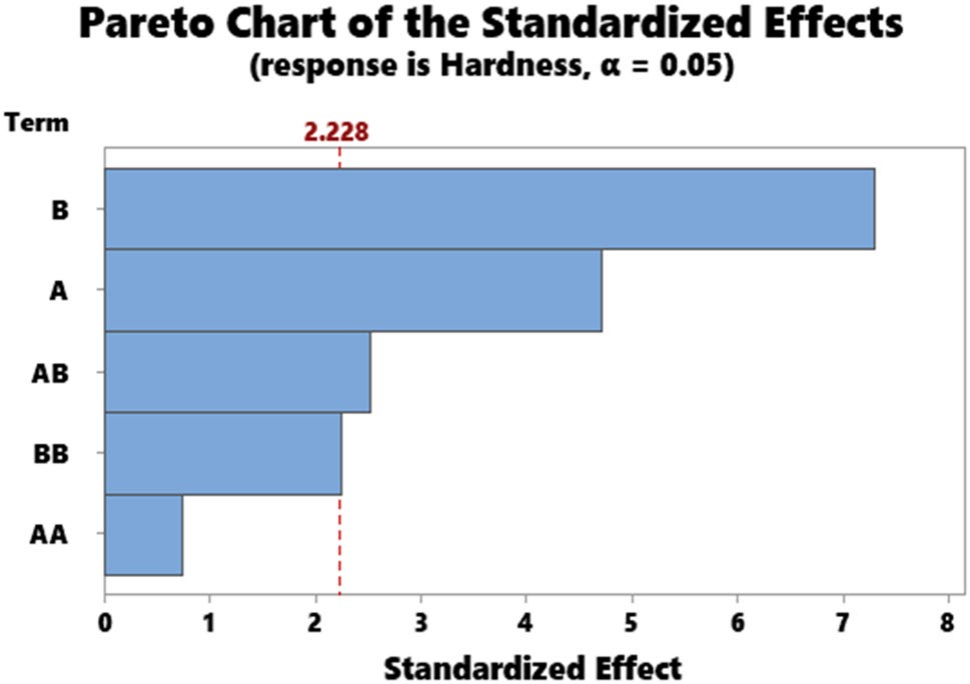
Pareto chart for hardness.

**Figure 8:**
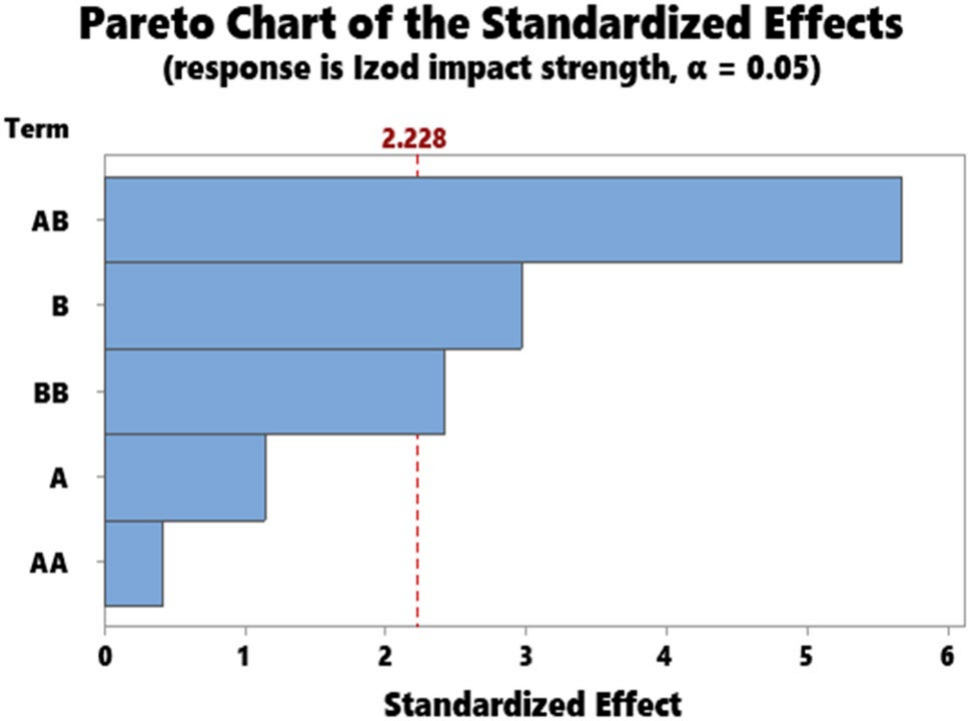
Pareto chart for impact strength.

**Figure 9:**
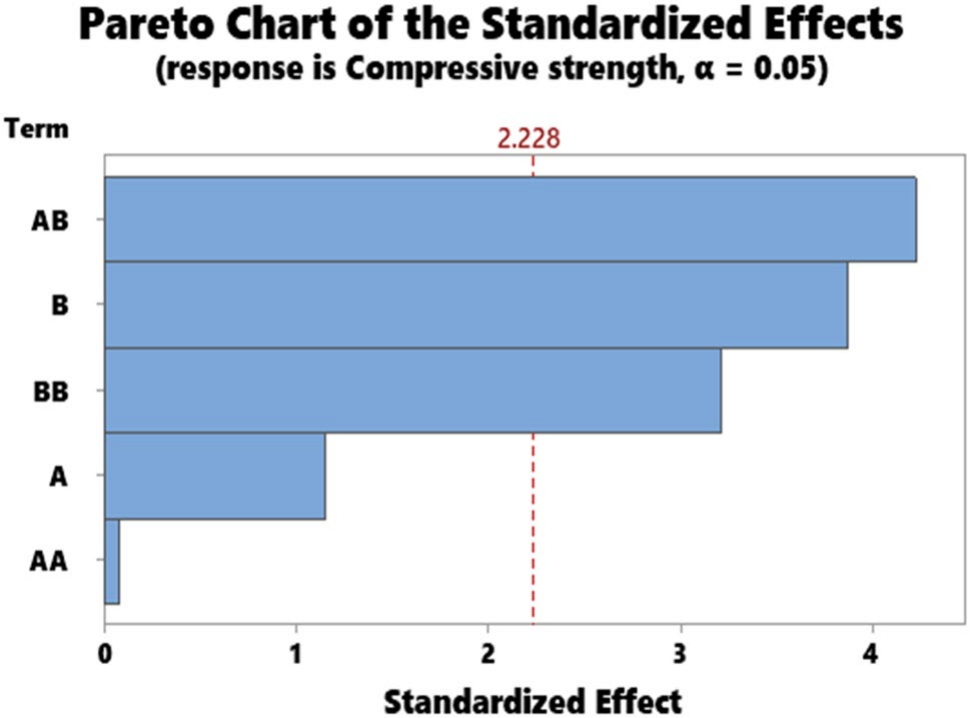
Pareto chart for compressive strength.

**Figure 10:**
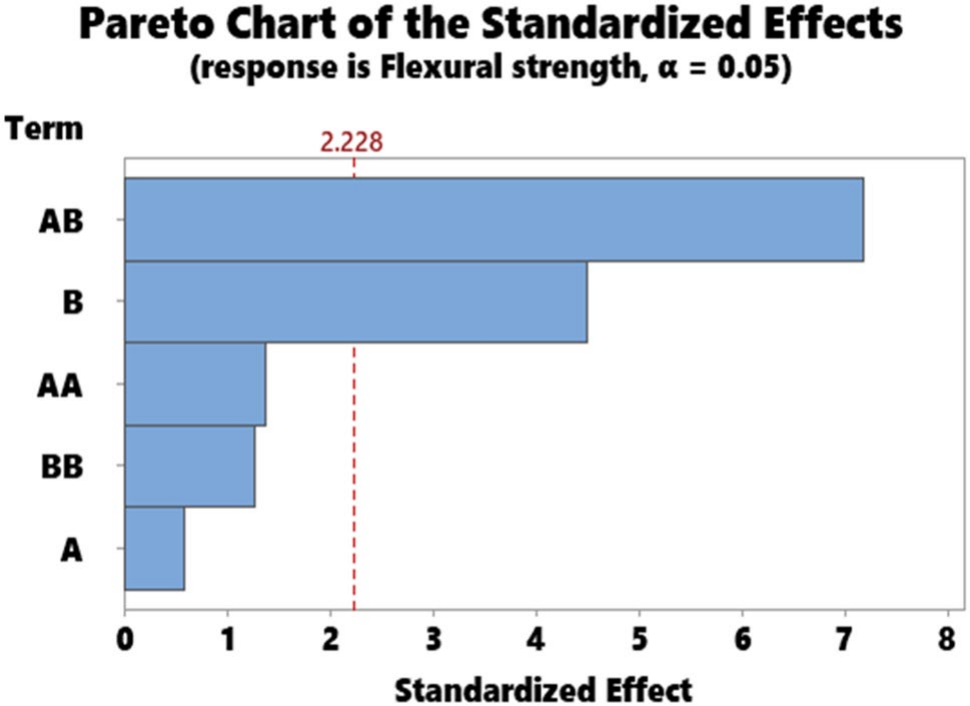
Pareto chart for flexural strength.

**Figure 11:**
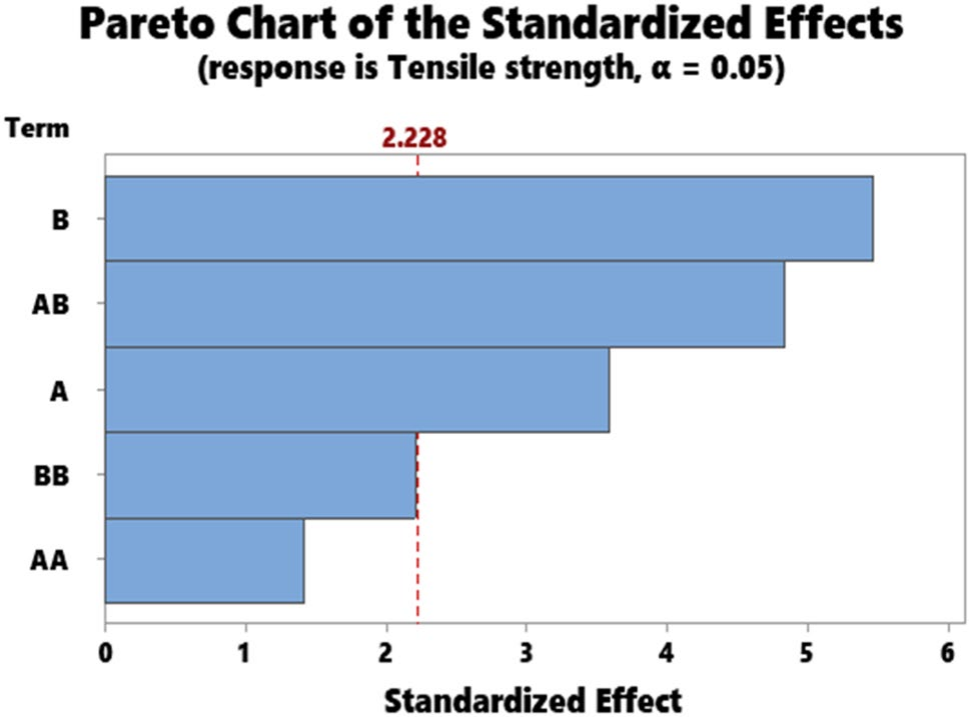
Pareto chart for tensile strength.

The representative results for the statistical data showing the ANOVA results for hardness, impact strength, compressive strength, flexural strength, and Tensile strength are as presented in Tables 3, 4, 5, 6, and 7 respectively. Accordingly, the Pareto chart for hardness, impact strength, compressive strength, flexural strength, and Tensile strength are as displayed in Figs. 7, 8, 9, 10, and 11 respectively.

**Table 4:** ANOVA for impact strength.

Source	DF	Adj SS	Adj MS	F-value	*P* value
A	1	35.67	35.674	1.32	0.278
B	1	238.52	238.523	8.81	0.014
A*A	2	237.57	118.787	4.39	0.043
B*B	1	4.84	4.844	0.18	0.681
A*B	1	158.59	158.593	5.86	0.036
Error	1	871.28	871.283	32.19	0.000
Total	10	270.70	27.070

**Table 5:** ANOVA for compressive strength.

Source	DF	Adj SS	Adj MS	F-value	*P* value
A	1	11.82	11.816	1.33	0.276
B	1	133.44	133.444	14.99	0.003
A*A	1	0.06	0.063	0.01	0.935
B*B	1	91.65	91.648	10.30	0.009
A*B	1	158.72	158.724	17.83	0.002
Error	10	89.02	8.902		
Total	15	1048.35

**Table 6:** ANOVA for flexural strength.

Source	DF	Adj SS	Adj MS	F-value	*P* value
A	1	2.56	2.565	0.33	0.576
B	1	155.18	155.182	20.24	0.001
A*A	1	14.52	14.516	1.89	0.199
B*B	1	12.23	12.230	1.60	0.235
A*B	1	394.72	394.723	51.48	0.000
Error	10	76.67	7.667		
Total	15	1186.86

**Table 7:** ANOVA for tensile strength.

Source	DF	Adj SS	Adj MS	F-value	*P* value
A	1	168.69	168.686	12.88	0.005
B	1	391.11	391.109	29.86	0.000
A*A	1	26.17	26.172	2.00	0.188
B*B	1	63.90	63.903	4.88	0.052
A*B	1	306.22	306.219	23.38	0.001
Error	10	130.98	13.098		
Total	15	2178.93

A represents kenaf fiber input (KF), B represents particulate snail shell input (PSS).

From Table 3, the P-value of inputs A and B are < 0.05 implying that at 95% confidence level, the contribution of KF and PSS on hardness was significant. Similarly, the interaction of A*B is significant while interactions A*A and B*B are insignificant at 95% confidence level. The order in which the parameters identified (A, B, and A*B) in the ANOVA table affect hardness is presented in the Pareto chart in (Fig. 7). The red line which points to 2.228 indicates the 95% confidence level, therefore, bars above line 2.228 depict parameters that play a significant role while the ones below, points to insignificant parameters. As observed in Fig. 7, parameter B is the most significant implication of which shows that particulate snail shells had a significant contribution to hardness. Next to PSS is Kenaf fiber followed by the interaction kenaf fiber *particulate snail shell.

Statistical analysis on impact strength is represented by ANOVA (Table 4) and Pareto chart (Fig. 8). From Table 4, parameters B, A*A, and A*B significantly affect impact strength at the confidence level of 0.95 or 95% while parameters A and B*B had an insignificant effect on the strength. Figure 8 which highlighted the order of magnitude of the parameters based on frequency reveals particulate snail shell is the most significant input. Kenaf fiber * particulate snail shell interaction is next followed by kenaf fiber. Parameters B*B and A*A are insignificant.

As for compressive strength, contributions of particulate snail shell and interaction B*B, A*B had a significant effect on compressive strength (Table 5). Parameters A and A*A had no significant influence on the strength. The order of importance of the parameters according to (Fig. 9) are AB, B, and BB for significant parameters. Parameters A and AA are under the confidence level line 2.228, therefore had insignificant influence on strength value. Analysis of ANOVA for flexural strength (Table 6) showed that parameters B and AB significantly influence the value of flexural strength while the order of influence is AB and A according to the Pareto chart for compressive strength (Fig. 10).

Parameters A, B, and A*B significantly influence tensile strength as depicted in (Table 6) while AA and BB displayed the insignificant influence of tensile strength. Meanwhile, the Pareto chart detected that B, AB, and A are in the order of the significant parameters (Fig. 11).

### Taguchi analysis on each property

**Figure 12:**
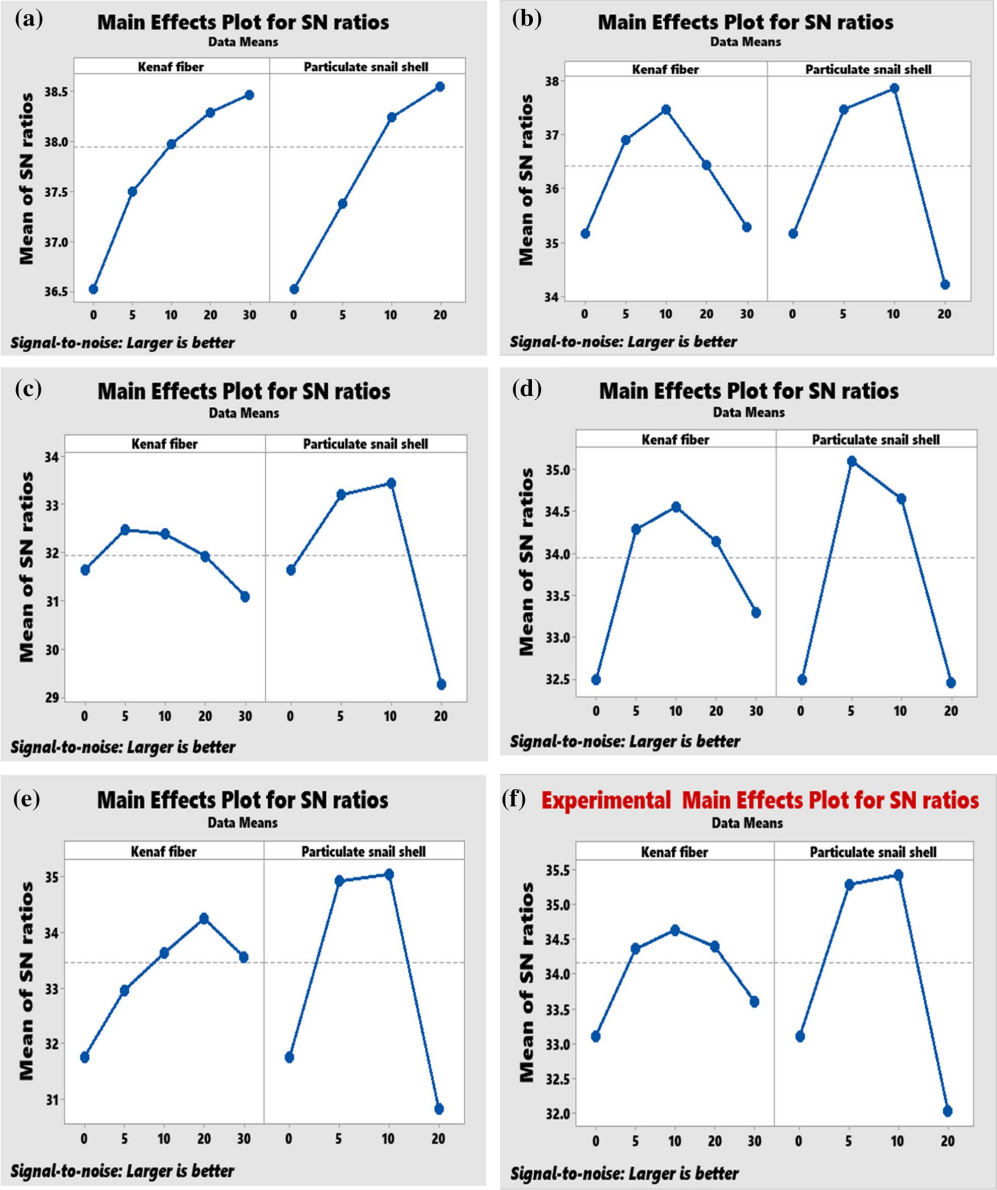
Taguchi optimization.

The various trend of the Taguchi analysis (main effect plot for S/N ratios) on the experimental results is presented in Fig. 12a–f respectively.

Figure 12a reflects Taguchi's report on hardness from which the combination of 30 wt% KF and 20 wt% PSS gave an optimal result for the property. As observed in Fig. 12b, the combination of 10% KF and 10% PSS yielded optimum impact strength. Optimum for compressive, flexural, and tensile strengths are the combination of 10% KF and 4% PSS, 10% KF and 2% PSS, and 20% KF and 4% PSS.

Taguchi optimization was carried out simultaneously on hardness, impact strength, compressive, flexural, and tensile strengths using Minitab 19 application. It was applied to determine input parameters that will yield optimum performance for all properties. Figure 12f shows that kenaf fiber at 10 wt% and particulate snail shell at 4 wt% respectively shows optimum combined properties.

## Conclusions

Kenaf fiber (KF) and particulate snail shell (PSS) were added to polypropylene (PP) matrix with the view of improving the mechanical properties for engineering application, the following conclusions were arrived at;

1. the blend of 2 wt% PSS and 5, 10, 20, and 30 wt% KF ensued progressive enhancement of hardness, impact, compressive and flexural strength with 2 wt% PSS/30 wt% yielding 14.9, 46.4, 27.5, and 53.4% for each property respectively. For flexural strength, optimum improvement was attained when 2 wt% PSS was combined with 30 wt% KF. Meanwhile, tensile strength achieved an optimum improvement of 72.9% when 2 wt% PSS was combined with 20 wt% KF.
2. presence of 4 wt.% PSS, 5, 10, 20, and 30 wt% KF engendered progressive enhancement of hardness, 4 wt% KF yielded 29.5 optimum improvements for impact strength while the same dosage yielded optimum enhancement of 36.9% over neat PP for compressive strength. Flexural strength was enhanced by admix of 5 and 10 wt% KF even as further addition of 20 and 30 wt% KF led to a depreciation in strength. Tensile strength showed progressive enhancement up to 20 wt% KF before a further decline.
3. the blend of 10 wt% PSS and 5, 10, 20 and 30 wt% KF triggered enhancement of hardness, the same proportion spawned downward trend in impact, compressive, flexural, and tensile strength as proportion KF increased from 5 to 30%. Hence, intermix of 10 wt% PSS and kenaf fiber from 5 to 30 wt% are detrimental to properties of PP matrix.
4. Taguchi optimization revealed an optimum mix proportion of 4 wt% PSS/10 wt% KF. Conclusively, particulate snail shell derived from African giant snail and kenaf bast fiber are good reinforcement in polypropylene in the development of a  cost effective but stronger biocomposites which can find applications in automobile and aerospace application.
5. developed composite in this study showed better performance than their counterpart reinforced with hydroxylapatite and chitosan which are mostly used in reinforcement of biopolymers.

## Data Availability

All data generated or analyzed during this study are included in this published article.

## Code availability

No code was used for the computation of the data reported in this study.
